# The mental health impact of school bullying among young carers in Australia: a causal mediation analysis

**DOI:** 10.1038/s41598-023-43464-5

**Published:** 2023-10-05

**Authors:** Ludmila Fleitas Alfonzo, Ankur Singh, George Disney, Tania King

**Affiliations:** 1 Disability and Health Unit, School of Population and Global Health, Centre for Health Equity, The University of Melbourne, Level 4, 207, Bouverie Street, MelbourneParkville, VIC 3010 Australia; 2 Centre of Epidemiology and Biostatistics, Melbourne School of Population and Global Health, The University of Melbourne, Parkville, VIC 3010 Australia

**Keywords:** Psychology, Public health, Epidemiology

## Abstract

Informal care can exert adverse effects on the mental health of young people. Bullying victimisation is an important determinant of mental disorders. Young carers are at elevated risk of bullying. We quantify the mental health effects of informal care among Australian adolescents and the extent to which these effects are transmitted through school bullying. We used data from the Longitudinal Study of Australian Children. Participants were classified as non-carers, light carers (caring for < 10 h/week) and moderate-to-heavy carers (caring for 10 + h/week). Mental health was measured using the Kessler Psychological Distress Scale (K10). Using a counterfactual approach to mediation analysis, total effects (TE) of informal care on mental health were decomposed into natural direct effects (NDE—mental health effects not transmitted through school bullying) and natural indirect effects (NIE—mental health effects transmitted through school bullying). The TE of informal caring was 0.71 (95%CI − 0.03, 1.49) for light carers and 1.72 (95%CI 0.45, 3.02) for moderate-to-heavy carers. While school bullying explained 27% of the TE among moderate-to-heavy carers (NIE: 0.46; 95%CI 0.12, 0.91) there was weak evidence of mediation for light carers. Our findings indicate that the mental health effects of moderate-to-heavy caregiving can be reduced by school bullying interventions.

## Introduction

In Australia, informal carers provided approximately 2.28 billion hours of care in 2020, with an estimated replacement cost of 77.9 billion dollars^1^. This contribution was partly shouldered by young informal carers^1^. Global estimates suggest that 2–8% of the population aged 12–25 years provide unpaid support towards a sibling, parent or other relatives in need of regular assistance^2^. Due to the increasing prevalence of chronic diseases and population aging, it is anticipated that informal care needs are likely to increase^1^, with more young people taking on these roles.

Informal care exerts negative impacts on the psychological well-being of middle-aged adults^3–6^. Longitudinal evidence suggests that informal care increases the risk of depressive symptoms^4^, anxiety^3^, and other mental health problems^6^ among adults. A large body of qualitative research indicates that young informal carers also experience negative outcomes for undertaking these roles^7–9^. For instance, they are likely to report experiences of isolation^8^, stigma^9^, and emotional distress^8^.

A recent systematic review identified that although longitudinal studies of the effects of informal care on the mental health among young people are still scarce, findings across these studies consistently demonstrate a negative effect^10^. Using a sample of Australian adolescents, King et al.^11^ found that informal care at the age of 14–15 years resulted in an increased risk of psychological distress four years later. These results align with studies in the UK, where other longitudinal studies found that young informal carers are more likely to display poor mental health^12^, higher psychological distress^13^ and poorer psychological wellbeing^13^ than their non-caring peers. While findings from these studies establish a causal effect of informal care on youth mental health, the causal pathways through which such effects occur are not clear. Given that most mental health disorders start in adolescence and youth^14^, informal caring may place young carers at increased risk of poor psychological well-being in later adulthood. Thus, determining the underlying mechanisms explaining this causal relationship is imperative to providing evidence-based intervention strategies to support young carers’ mental health.

Bullying victimisation is a key determinant of poor mental health in youth^15^, and young carers may be particularly susceptible to the mental health effects of bullying^16^. Qualitative research suggests that experiences of peer victimisation and harassment are barriers to school participation among young carers^9^. This is especially important because, for many young carers, schools are the only place in which they can disconnect from their caring demands^9^. Moreover, informal care can foster feelings of social isolation in adolescent carers as they perceive themselves as being different from their peers^7^. Peer bullying may exacerbate these feelings, further isolating young carers^16^ and increasing the psychological distress associated with informal care.

Therefore, it is plausible that the mental health impact of informal care is partially transmitted through experiences of school bullying victimisation. This paper has two aims: (i) to quantify the total effects of informal care on the mental health of Australian adolescents and (ii) to examine the extent to which bullying victimisation at school mediates these effects.

## Methods

We conducted a prospective analysis of secondary data from the Longitudinal Study of Australian Children (LSAC).

### Study population

LSAC is a nationally representative sample of Australian children and youth that has followed two cohorts of children since 2004. Participants in cohort B were aged 0–1 years at baseline while those in cohort K were 4–5 years old. A two-stage sample design was used to sample eligible participants. In stage one, 311 postcodes were selected and stratified by states and territories to ensure proportional representation of each Australian region. Eligible children living in these postcodes were randomly selected using the Medicare database^17^. LSAC data is collected biennially, and data sources include the study child, parents living with the child and elsewhere, and teachers or childcare workers^18^.

Our analyses focused on participants of cohort K, for whom data on young caring activities were collected for the first time in 2014 (study child aged 14/15 years). Cohort B was excluded because information on informal care is only available for wave 8. In order to ensure the temporal ordering of exposure, mediator and outcome, data was extracted from waves 5 (2012), 6 (2014), 7 (2016) and 8 (2018); participants’ ages 12/13, 14/15, 16/17, 18/19 years, respectively^18^.

### Exposure: informal care

Data on informal caring was extracted from wave 6. Data sources included the study child and their parents. In the survey, all children were asked: “*Do you help someone who has a long-term health condition, has a disability or is elderly, with activities that they would have trouble doing on their own*”*.* Another question was asked to parents of children living with someone with a disability “*Thinking about [person] and his/her [medical condition/restriction] does the Study Child help them with everyday activities?*”*.* We combined responses to both questions to identify young carers in the sample. Using responses from different sources allowed us to mitigate the risk of misclassification of the exposure given that many young carers may not identify themselves as such^19^. A follow-up question determined the frequency of caring activities. Response options included “*Every day*”, “*At least once a week*”, “*At least once a fortnight*”, “*At least once a month*” and “*Less than once a month*”. Young carers and their parents were also asked to indicate how much time the study child spent on caring activities. Response options included “*Less than 2 h*”, “*2 to less than 5 h*”, “*5 to less than 10 h*”, “*10 to less than 15 h*”, “*15 to less than 20 h*” and “*20 h or more*”. We created a categorical variable to reflect caring status as well as the frequency and extent of caregiving activities. Participants were classified as non-carers, light carers (caring for < 10 h/week, only 1/fortnight or 1/month) and moderate-to-heavy carers (caring daily or 10 + h/week). This definition aligns with that of Colombo et al.^20^, who classify informal caring demands as low (< 10 h of weekly care), medium (10–19 h of weekly care) and high intensity (20 + hours of weekly care). Given the small number of adolescents undertaking a high intensity of caring demands, young carers providing medium and high intensity caregiving were grouped under the moderate-to-heavy category.

### Outcome: psychological distress

Mental health status in wave 8 of LSAC was measured using the Kessler Psychological Distress Scale (K10)^21^, a validated measure of psychological morbidity. High K10 scores are strongly associated with a diagnosis of anxiety, depression and other mental disorders^22^. Participants were asked 10 questions reflecting experience of anxiety and depression in the past four weeks. Response options ranged from 1 “*none of the time*” to 5 “*all of the time*”. Total scores for all questions were used as a continuous measure, with higher scores indicating poorer mental health.

### Mediator: school bullying

Data on experiences of bullying victimisation were extracted from wave 7 as reported by the study child. Nine items denoting bullying victimisation in the past month were measured (see Supplementary Table 1). Participants were also asked about the location where these experiences took place. A binary variable was generated to represent experiences of bullying victimisation at school (yes/no). Therefore, the term “school bullying” throughout this paper reflects victimisation and is not inclusive of experiences of perpetration.

### Confounding factors

Potential confounding factors were extracted from wave 5. These included gender, quintiles of weekly household income, parental employment (both parents employed, one parent employed, both parents unemployed), maternal highest educational qualification (certificate/diploma, year 12, less than year 12), parental cultural background (both parents born in Australia, at least one parent born in Anglo-English speaking country, at least one parent born in Non-English speaking country, at least one parent identifies as Indigenous Australian), both parents in household (yes, no), children under five years in household (yes, no), number of siblings in household and area-level disadvantage (quintiled). To account for the confounding effect of parental/family illness^23, 24^, commonly referred to as the “family effect” in the informal care literature^25^, all of our models were adjusted for the presence of a family member with a disability or health condition. This variable was reported by Parent 1 (often the mother) and defined as having a household member with a disability (yes, no). The variables listed above were identified as common causes of informal care, school bullying and mental health. Supplementary Fig. 1 displays a directed acyclic graph representing our confounding assumptions. While parental employment and household income are likely to vary over time, preliminary analyses indicated that the distribution of these variables did not change substantially across LSAC waves 5–6 (see Supplementary Table 2) and, therefore, we only adjust for parental employment and household income as time invariant confounders. We note that gender could act as an effect modifier of the relationship examined. Specifically, gender may modify the extent of mediation through school bullying. Due to the sample size, we could not disaggregate the analysis across strata of boys and girls. Therefore, we only account for this factor as a potential confounder.

### Statistical analysis

We used a counterfactual approach to mediation analysis to estimate total effects (TE) of informal care on mental health, and decompose TE into natural direct effects (NDE, effect not transmitted through school bullying) and natural indirect effects (NIE, effect transmitted through school bullying). This approach was preferred over other mediation approaches as it allows for exposure-mediator interaction as well as the control of mediator-outcome confounding under the absence of exposure induced mediator-outcome confounding.^26, 27^.

We calculated K10 scores for three counterfactuals:

- E[Y(A(0), M(0)], representing the expected mental health scores for the unexposed (non-carers) with mediator (school bullying) values observed among the unexposed.
- E[Y(A(1), M(0)], denoting the expected mental health scores for the exposed (young carers) with mediator values observed among the unexposed.
- E[Y(A(1), M(1)], indicating the expected mental health scores for the exposed with mediator values observed among the exposed.

These counterfactuals were estimated using predicted mental health scores from two multivariable linear regression models. In the first model, informal care was regressed on mental health scores, adjusting for confounding factors. In the second model, an interaction term between informal care and school bullying was included. In order to answer our first aim, total effects of informal care on mental health scores were estimated by contrasting two counterfactuals E[Y(A(1), M(1)]) − E[Y(A(0), M(0)]. For the second aim, NDE and NIE were decomposed using a direct–indirect approach to effect decomposition^28^. NDE were calculated as the contrast between E[Y(A(1), M(0)] − E[Y(A(0), M(0)], while NIE were estimated as the difference between E[Y(A(1), M(1)] − E[Y(A(1), M(0)]. The proportion of mental health effects mediated through school bullying was calculated as the NIE divided by the TE. Bootstrapping with 1000 replications was used to calculate 95% confidence intervals.

Two separate analyses were conducted with non-carers as the reference group, one to assess the mental health effects of light caring and the second examining the effects of moderate-to-heavy caring. All analyses were performed using the complete case dataset. For each analysis, marginal TE, NDE and NIE were calculated, and confounding controlled for, using inverse probability weighting. These weights were estimated by generating propensity scores from a model regressing the exposure on potential confounding factors.

### Missing and non-response

A total of 3375 participants reported data on informal care in wave 6. Most of the non-response in wave 7 and 8 was due to sample loss—that is, respondents did not participate in the wave. A total of 2884 eligible adolescents participated in wave 7 and 2780 in wave 8 reported mental health data. After deleting participants who did not participate in all waves and those with missing data, the total number of participants with complete data was 2078. Supplementary Table 2 displays the proportion of missing data on mental health, school bullying and covariates by exposure to informal care.

### Multiple imputation

We performed multiple imputation using chained equations to address selection bias due to attrition and non-response. All covariates and the mental health variable were included in the model together with the following ancillary variables extracted from wave 1: number of siblings, area of remoteness, birth plurality, presence of two parents in household and parental relationship to the child. In order to combine the imputed and bootstrapped estimates, we used the MI-boot method^29^, in which the imputed coefficients were retrieved using the Stata command “mim” and bootstrapped with 1000 replications.

### Sensitivity analysis

We carried out two sets of sensitivity analyses: First, an additional set of models were adjusted for prior mental health, measured using scores of the Strengths and Difficulties Questionnaire (SDQ) and prior school bullying (yes, no). These were identified as potential common causes of experiences of school bullying and mental health status. Since the start of caring activities could not be ascertained, prior school bullying and mental health are also likely mediators of the association between informal care at ages 14/15 and mental health at 18/19 years. Therefore, this analysis was approached as a sensitivity test. Second, all analyses were repeated on the imputed sample.

### Ethics declaration

LSAC was approved by the Australian Institute of Family Studies Ethics Committee. LSAC meets the ethical standards highlighted in the National Statement on Ethical Conduct in Research Involving Humans, ensuring its performance fulfils the ethical requirements set on the Declaration of Helsinki.

The present project uses secondary data from LSAC and poses minimal risk to participants. However, independent ethics approval was sought and obtained from the Office of Research Ethics and Integrity at the University of Melbourne. Reference number 2021-20333-16440-3.

### Informed consent

Informed consent was obtained from the study child and their parents or legal guardians for data collection. Participation in LSAC was voluntary and participants could withdraw from the study or choose not to respond some parts of the survey.

### Role of the funding source

The funder was not involved in the conception of this paper, the data acquisition and analysis, interpretation of data, the preparation of the manuscript or in the decision to publish this article.

## Results

**Figure 1:**
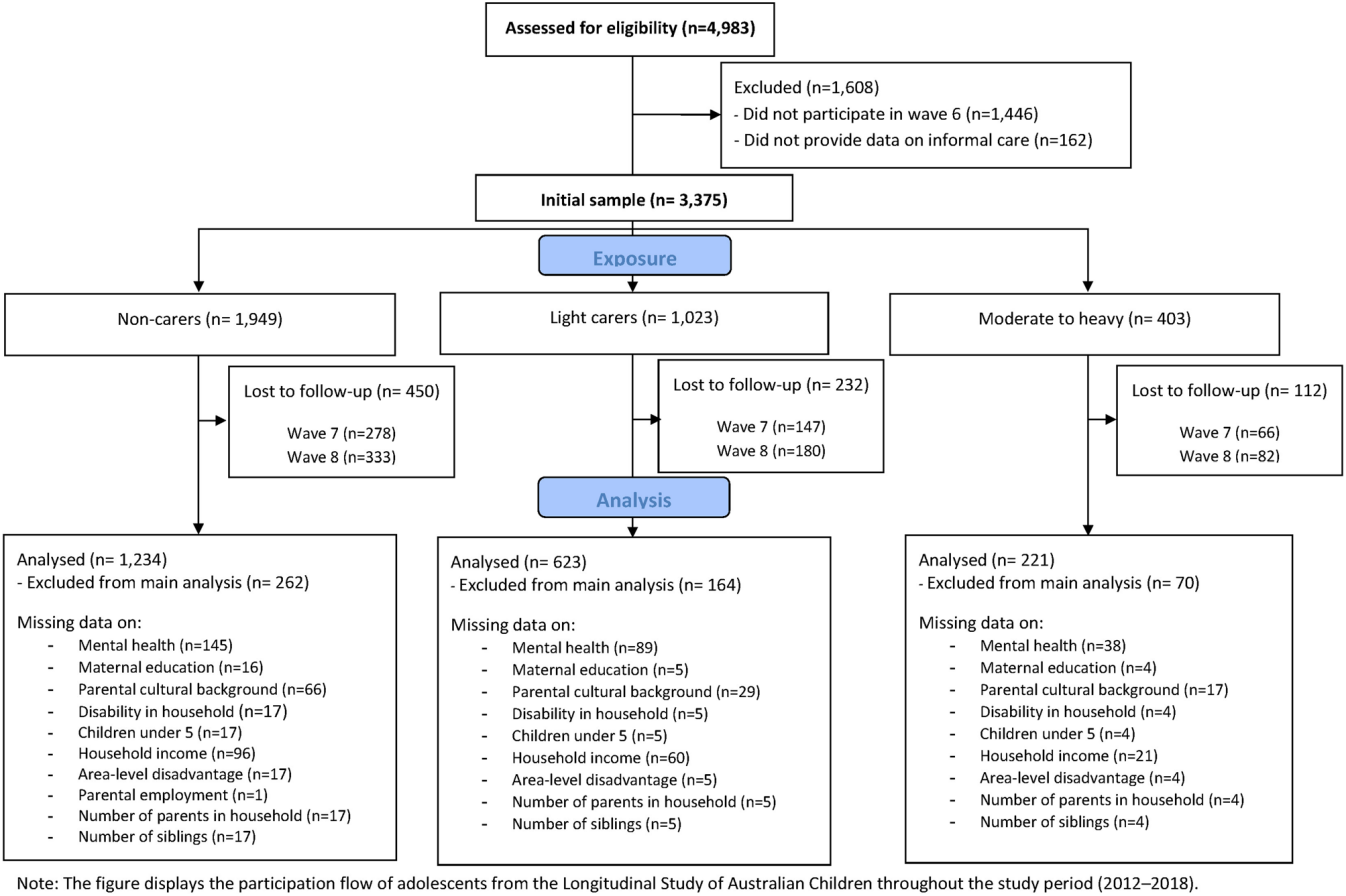
Participants flowchart.

Figure 1 displays participation flow of eligible participants by exposure status. Table 1 shows the distribution of mental health scores, school bullying and covariates by categories of informal care for participants in the complete case sample (n = 2078). The majority of participants had non-Indigenous Australian parents and lived with both parents at home. A higher proportion of males than females provided light caregiving, while gender distribution was similar for moderate-to-heavy carers. Mental health scores were higher for informal carers, as was their experience of school bullying. A higher proportion of carers than non-carers lived with children aged under five years and with someone with a disability. Prior experiences of school bullying were more frequent among carers and prior mental health scores were slightly higher for both informal caring categories than among non-carers.

**Table 1:** Distribution of outcome (mental health at 18/19–2018), mediator (school bullying at age 16/17–2016) and covariates (at age 12/13–2012) by exposure (informal caring at age 14/15–2014) in the complete case sample of Australian adolescents.

	Non-carers	Young carers		All
		Light	Moderate/heavy	
No. (%)	1234 (59.4)	623 (30.0)	221 (10.6)	2078 (100.0)
Outcome				
Kessler 10 scores (range, 10–50; mean (SD))	19.0 (7.70)	19.7 (8.41)	21.0 (9.13)	19.4 (8.10)
Mediator				
School bullying	No. (%)	No. (%)	No. (%)	No. (%)
No	971 (78.6)	469 (75.3)	153 (69.2)	1,592 (76.6)
Yes	264 (21.4)	154 (24.7)	68 (30.8)	486 (23.4)
Covariates				
Gender				
Males	591 (47.9)	348 (55.9)	109 (49.3)	1,048 (50.4)
Females	643 (52.1)	275 (44.1)	112 (50.7)	1,030 (49.6)
Parental cultural background				
Both parents born in Australia	829 (67.2)	422 (67.7)	158 (71.5)	1,409 (67.8)
At least one parent born in Anglo-English-speaking country	214 (17.3)	106 (17.0)	31 (14.0)	351 (16.9)
At least one parent born in Non-English-speaking country	172 (13.9)	82 (13.2)	28 (12.7)	282 (13.6)
Maternal highest educational qualification				
Certificate/diploma	400 (32.4)	207 (33.2)	74 (33.5)	681 (32.7)
Year 12	157 (12.7)	60 (9.63)	27 (12.2)	244 (11.7)
Less than year 12	677 (54.9)	356 (57.1)	120 (54.3)	1,153 (55.5)
Weekly household income				
(Lowest) 1	178 (14.4)	118 (18.9)	46 (20.8)	342 (16.4)
2	244 (19.8)	111 (17.8)	49 (22.2)	404 (19.4)
3	257 (20.8)	134 (21.5)	45 (20.4)	436 (21.0)
4	245 (19.8)	126 (20.2)	49 (22.2)	420 (20.2)
(Highest) 5	310 (25.1)	134 (21.5)	32 (14.5)	476 (22.9)
Area level of disadvantage (SEIFA)				
(Most deprived) 1	241 (19.5)	127 (20.4)	63 (28.5)	431 (20.7)
2	233 (18.9)	106 (17.0)	42 (19.0)	381 (18.3)
3	263 (21.3)	139 (22.3)	50 (22.6)	452 (21.8)
4	243 (19.7)	122 (19.6)	34 (15.4)	399 (19.2)
(Least deprived) 5	254(20.6)	129 (20.7)	32 (14.5)	415 (20.0)
Both parents in household				
Yes	1084 (87.9)	522 (83.8)	196 (88.7)	1,802 (86.7)
No	150 (12.1)	101 (16.2)	25 (11.3)	276 (13.3)
Children under 5 in household				
No	1148 (93.0)	576 (92.5)	196 (88.7)	1,920 (92.4)
Yes	86 (6.97)	47 (7.54)	25 (11.3)	158 (7.60)
Household member with a disability				
No	885 (71.7)	360 (57.8)	84 (38.0)	1,329 (63.9)
Yes	349 (28.3)	263 (42.2)	137 (62.0)	749 (36.1)
Siblings in household (range, 0–8; mean(SD))	1.52 (0.94)	1.53 (1.07)	1.76 (1.08)	1.54 (1.00)
Parental employment				
Both parents employed	887 (71.8)	423 (67.9)	128 (57.9)	1,438 (69.2)
One parent employed	309 (25.0)	162 (26.0)	71 (32.1)	542 (26.1)
Both parents unemployed	38 (3.08)	38 (6.10)	22 (9.95)	98 (4.72)
Prior school bullying				
No	914 (76.1)	440 (72.4)	127 (59.6)	1,481 (73.3)
Yes	287 (23.9)	168 (27.6)	86 (40.4)	541 (26.8)
SDQ (range, 0–32; mean (SD))	8.33 (5.40)	9.17 (5.73)	9.64 (5.62)	8.72 (5.54)

Abbreviations: *SD* standard deviation, *SEIFA* Socio-Economic Indexes for Areas, *SDQ* strength, and difficulties scores. Note: Due to small cell numbers, the distribution of participants according to parental Indigenous background was omitted from this table.

Table 2 displays TE, NDE and NIE for the effects of informal care on Kessler scores of Psychological Distress (K10) mediated through school bullying. For light carers, the total effect of informal care on K10 scores was 0.71 (95%CI − 0.03, 1.49), with weak evidence of mediation by school bullying (NIE: 0.10; 95%CI − 0.05, 0.27); this accounting for 13% of the effect of informal care on mental health. The total effect of informal care on K10 scores was higher for moderate-to-heavy carers (TE: 1.72; 95%CI 0.45, 3.02). This effect was partly mediated by school bullying (27%) with a NIE of 0.46 (95%CI 0.12, 0.91).

**Table 2:** Estimates of natural indirect effects and natural direct effects of the association between informal care and mental health, mediated by bullying victimisation (Australia. 2012–2018).

	Total effect	Natural direct effect	Natural indirect effect^a^	Proportion mediated
Light carers	*ß (95%CI)*	*ß (95%CI)*	*ß (95%CI)*	*Proportion (95%CI)*
Model 1^b^	0.71(− 0.03, 1.49)	0.41 (− 0.10, 1.40)	0.10 (− 0.05, 0.27)	0.13 (− 0.30, 1.51)
Model 2^c^	0.54 (− 0.20, 1.25)	0.45 (− 0.29, 1.16)	0.09 (− 0.05, 0.25)	0.17 (− 1.12, 1.26)
Model 3^d^	0.54 (− 0.20, 1.25)	0.44 (− 0.28, 1.14)	0.10 (− 0.04, 0.26)	0.17 (− 0.79, 2.21)
Moderate-to-heavy carers				
Model 1^b^	1.72 (0.45, 3.02)	1.25 (0.02, 2.46)	0.46 (0.12, 0.91)	0.27 (0.08, 0.94)
Model 2^c^	1.38 (0.18, 2.68)	1.04 (− 0.09, 2.27)	0.34 (0.02, 0.78)	0.25 (− 0.01, 1.06)
Model 3^d^	1.38 (0.21, 2.65)	0.94 (− 0.23, 2.19)	0.45 (0.10, 0.90)	0.32 (0.03, 1.34)

^a^Effect mediated through school bullying.

^b^Adjusted for gender, parental cultural background, maternal highest educational qualification, weekly household income, area level of disadvantage, both parents in household, children under 5 in household, household member with a disability, siblings in household and parental employment.

^c^Adjusted for variables in model 1 and mental health at 12/13 years (Strength and difficulties scores).

^d^Adjusted for variables in model 2 and school bullying at 12/13 years.

### Sensitivity analyses

After adjusting for prior mental health (SDQ scores), the total effects of informal care on mental health at age 18/19 years were consistent with main analyses, although attenuated for light carers (TE: 0.54; 95%CI − 0.20, 1.25) and moderate-to-heavy carers (TE: 1.38; 95%CI 0.18, 2.68) (see Table 2). Total effects for light and moderate-to-heavy carers were not further attenuated by prior school bullying. Evidence of mediation did not substantially change for light carers with a NIE of 0.10 (95%CI − 0.04, 0.26) in models adjusted for both prior mental health and school bullying. Mediation was still present for moderate-to-heavy carers in models adjusted for prior mental health (NIE: 0.34; 95%CI 0.02, 0.78) and further adjusted for prior school bullying (NIE: 0.45; 95%CI 0.10, 0.90). Lastly, mediation through school bullying among moderate-to-heavy carers was also present in the imputed analysis (see Supplementary Table 4).

## Discussion

Our findings indicate that informal care has a detrimental effect on young carers' mental health. Mental health effects are stronger for young people providing a substantial amount of caregiving, indicating a dose–response relationship. Mediation of the mental health effects of informal care among Australian adolescents through school bullying was confirmed only for moderate-to-heavy carers. Our results were consistent across sensitivity tests adjusting for prior experiences of school bullying and prior mental health scores. These findings were also consistent across the complete case and imputed analyses.

In the context of recent systematic reviews on the mental health of young carers^10, 30^, this paper directly answers the need for more causally focused research on this association, a key gap in this area^10, 30^. Our results align with cross-sectional research demonstrating a negative association between young caring and mental health, with a clear dose–response relationship^31^, as well as other emerging longitudinal evidence supporting a causal effect of informal care on young people's mental health^11, 13^. One explanation for these findings relates to role overload theory, which posits that substantial caregiving demands, such as those undertaken by moderate-to-heavy carers, restricts the time and resources a caregiver possesses to manage multiple obligations^32^. This role overload can intensify existing mental health strains related to the caring role, placing young carers at high levels of psychological distress and leading to poor psychological symptomatology^32^. The substantial caring demands faced by many informal carers can also prevent young carers’ participation on social and leisure activities^7, 33^. The limited social support and opportunities for respite from their caregiving roles may compound feelings of apprehension and loneliness in young carers who often need external support to cope with the emotional impact of their demands^9^.

As mentioned in both systematic reviews, pathways through which young informal care leads to poor mental health outcomes have not been investigated^10, 30^. Although recent evidence postulates benefit finding (the ability to identify positive changes after experiencing adverse events) as a protective factor for the mental health of young caregivers^34^, no previous study has explored the underlying mechanisms explaining the poorer psychological health of adolescent carers compared to their non-caring peers. Bullying victimisation is a deleterious experience that can have lifelong effects for the individual^15^. Ours is the first study to examine school bullying as a mediator of the effect of informal care on adolescents' mental health. Our findings are consistent with qualitative evidence reporting that young carers' experiences of bullying victimisation in schools may exacerbate distress related to caring demands^35, 36^. We also found that a higher proportion of young carers than non-carers experience school bullying. This is consistent with qualitative research suggesting that bullying experiences in young carers are related to the caring role or discrimination against the individuals being cared for^36^. When related to the caring roles, school bullying could intensify feelings of dissonance in young carers, who already feel isolated from their peers^7^. This then, may place young carers at even higher risk of the harmful effects of bullying compared to other population groups.

These findings indicate that intervening in school bullying may reduce mental health inequalities between young carers undertaking moderate-to-heavy caring and their non-caring peers. While we are not aware of targeted school bullying interventions for young carers, universal anti-bullying interventions have been trialled in school environments^37^. A meta-analysis of these interventions estimated that general anti-bullying prevention strategies in schools could reduce the prevalence of bullying victimisation by 15–16%^37^, while another meta-analysis showed that universal bullying programs were at least as, if not more, effective than targeted programs^38^. Future research should explore the potential of these interventions in reducing school bullying experiences of young carers.

This study has many strengths. First, we used a representative sample of adolescents, increasing the external validity of our findings. Second, this paper uses longitudinal data, ensuring the temporal ordering between covariates, exposure to informal care, mediation by bullying victimisation and the mental health outcome. Third, we used a counterfactual approach to mediation analysis which allowed us to account for mediator-outcome confounding and interaction between informal care and school bullying. Fourth, we used different sources to ascertain adolescents' exposure to informal care. The combination of responses from parents' and adolescents' reports allowed us to minimize the potential risk of misclassification of exposure to informal care. Lastly, we used a highly validated measure of psychological morbidity.

However, our results should be considered in the context of some limitations. The substantial attrition from the initial sample may have introduced selection bias, although we addressed this limitation by repeating our main analysis using an imputed sample. Another limitation is the use of self- and parent-reported data. Differential measurement errors of mental health and school bullying were minimised since the study participants and their parents were unaware of the study's aims at the time of data collection. Non-differential measurement error of the outcome was mitigated by using a highly validated mental health measure. While the risk of non-differential misclassification of school bullying was uncontrolled for, this will likely attenuate the observed effects. Furthermore, our findings are only applicable to contexts similar to Australia and therefore not generalisable to low- and middle-income countries or contexts in which informal care is typically expected from adolescents.

Mental health effects of young caring could vary by the condition of the care recipient. Our analyses have not distinguished by care recipient and as such, our findings provide little information about differences in mental health effects related to the condition of the individual being cared for. Given that problems related to mental health conditions are often stigmatised and require a higher level of emotional support, the effect of young informal care on bullying victimisation and mental health attributed to supporting a relative with specific support needs may be masked in our analyses. We recommend that future research interrogates variations in mental health effects of young caring, and mediation through school bullying, according to the individual conditions and support needs of the care recipient.

Our results may also differ by gender. While all our analyses account for gender as a confounding factor, there is some possibility that gender acts as an effect modifier of the relationship between informal care and mental health. Due to the limited sample size of informal carers in the sample, we could not disaggregate our analyses across strata of boys and girls. We recommend that future research explores this avenue. We also note that as opposed to Australian^39^ and other international estimates^34^, the gender split of informal care in LSAC is similar for boys and girls. This difference might be due to variations in the wording of caregiving questions. For example, in Australia, the Survey of Disability Aging and Carers asks participants whether a household member supports someone with specific everyday activities (such as communication, mobility and personal care)^39^. On the other hand, the LSAC identification question does not list any potential caring tasks. Given that boy carers may underreport their caregiving roles due to normative beliefs around gender and care^19^, the use of a broader question in LSAC may have led boy carers to report their caregiving to a greater extent than in other measures. The gender distribution of informal care identified here, however, aligns with US estimates on prevalence of informal care among young carers aged 8–18 years^40^.

Nonetheless, our findings have important implications for the mental wellbeing of adolescent carers. First, partial mediation could suggest that other pathways (such as access to support services, including respite care, school, and social support) may be of importance. We recommend that future research should explore these and other pathways. More importantly, over a quarter of the mental health effects of informal care among moderate/heavy carers were explained through school bullying, meaning that reducing its prevalence among adolescent carers could decrease the mental health inequalities between young carers and their non-caring peers. Considering that adolescence is a key period in development, and that most mental disorders start at youth, interventions to mitigate the mental health impacts of informal care in adolescence could have lasting effects over the life course. Given our findings and previous evidence on the harmful effects of bullying, preventing school bullying may deliver benefits for adolescents undertaking considerable caring demands.

### Supplementary Information

Supplementary Information.

## Data Availability

The data that support the findings of this study are available from the Australian Data Archive (ADA), through the ADA dataverse, but restrictions apply to the availability of these data, which were used under license for the current study, and so are not publicly available. Data are however available from the authors upon reasonable request and with permission of the Australian Data Archive and the National Centre of Longitudinal Data.

## References

[1] Deloitte Access Economics. The value of informal care in 2020. Report for Carers Australia., (Canberra, 2020).

[2] Becker S (2007). Global perspectives on children’s unpaid caregiving in the family: Research and policy on “young carers” in the UK, Australia, the USA, and Sub-Saharan Africa. Global Social Policy.

[3] Cannuscio CC (2002). Reverberations of family illness: A longitudinal assessment of informal caregiving and mental health status in the Nurses’ Health Study. Am. J. Public Health.

[4] Bijnsdorp FM (2022). Associations of combining paid work and family care with gender-specific differences in depressive symptoms among older workers and the role of work characteristics. Scand. J. Work Environ. Health.

[5] Bom J, Stöckel J (2021). Is the grass greener on the other side? The health impact of providing informal care in the UK and the Netherlands. Soc. Sci. Med..

[6] Lacey RE, McMunn A, Webb E (2019). Informal caregiving patterns and trajectories of psychological distress in the UK Household Longitudinal Study. Psychol. Med..

[7] Stamatopoulos V (2018). The young carer penalty: Exploring the costs of caregiving among a sample of Canadian youth. Child Youth Serv..

[8] Kavanaugh MS, Noh H, Studer L (2014). “It’d be nice if someone asked me how I was doing. Like, ‘cause I will have an answer”: exploring support needs of young carers of a parent with Huntington’s disease. Vulnerable Child. Youth Stud..

[9] Moore T, McArthur M, Noble-Carr D (2010). Different but the same? Exploring the experiences of young people caring for a parent with an alcohol or other drug issue. J. Youth Stud..

[10] Fleitas Alfonzo L, Singh A, Disney G, Ervin J, King T (2022). Mental health of young informal carers: A systematic review. Soc. Psychiatry Psychiatr. Epidemiol..

[11] King T, Singh A, Disney G (2021). Associations between young informal caring and mental health: A prospective observational study using augmented inverse probability weighting. Lancet Reg. Health Western Pacific.

[12] Brimblecombe N, Knapp M, King D, Stevens M, Cartagena Farias J (2020). The high cost of unpaid care by young people: Health and economic impacts of providing unpaid care. BMC Public Health.

[13] Nakanishi M (2022). Adolescent carers' psychological symptoms and mental well-being during the COVID-19 pandemic: Longitudinal study using data from the UK Millennium Cohort Study. J. Adolesc. Health.

[14] de Girolamo G, Dagani J, Purcell R, Cocchi A, McGorry PD (2012). Age of onset of mental disorders and use of mental health services: Needs, opportunities and obstacles. Epidemiol. Psychiatr. Sci..

[15] Moore SE (2017). Consequences of bullying victimization in childhood and adolescence: A systematic review and meta-analysis. World J. Psychiatry.

[16] Lindley, S. & Phelps, D. *Protecting Young Carers from Bullying. A Guide for Schools, Community Groups and Policy Makers* (London, 2016).

[17] Soloff, C., Lawrence, D. & Johstone, R. Longitudinal Study of Australian Children technical paper no. 1: sample design. Australian Institiue of Family Studies, Melbourne. (2006).

[18] Mohal, J. *et al.* (Australian Institute of Family Studies, Melbourne 2022).

[19] Smyth C, Blaxland M, Cass B (2010). ‘So that's how I found out I was a young carer and that I actually had been a carer most of my life’. Identifying and supporting hidden young carers. J. Youth Stud..

[20] Colombo, F., Llena-Nozal, A., Mercier, J. & Tjadens, F. Help Wanted?: Providing and Paying for Long-Term Care. *OECD Health Policy Studies*. 10.1787/9789264097759-en (2011).

[21] Kessler RC (2002). Short screening scales to monitor population prevalences and trends in non-specific psychological distress. Psychol. Med..

[22] Andrews G, Slade T (2001). Interpreting scores on the Kessler Psychological Distress Scale (K10). Austr. N. Z. J. Public Health.

[23] Pakenham KI, Cox S (2015). The effects of parental illness and other Ill family members on the adjustment of children. Ann. Behav. Med..

[24] Cassidy T, Giles M, McLaughlin M (2014). Benefit finding and resilience in child caregivers. Br. J. Health Psychol..

[25] Bom J, Bakx P, Schut F, van Doorslaer E (2019). Health effects of caring for and about parents and spouses. J. Econ. Ageing.

[26] VanderWeele T (2015). Explanation in Causal Inference: Methods for Mediation and Interaction.

[27] VanderWeele TJ (2016). Mediation analysis: A practitioner's guide. Annu. Rev. Public Health.

[28] Nguyen TQ, Schmid I, Stuart EA (2020). Clarifying causal mediation analysis for the applied researcher: Defining effects based on what we want to learn. Psychol. Methods.

[29] Schomaker M, Heumann C (2018). Bootstrap inference when using multiple imputation. Stat. Med..

[30] Lacey RE, Xue B, McMunn A (2022). The mental and physical health of young carers: A systematic review. Lancet Public Health.

[31] Tseliou F, Rosato M, Maguire A, Wright D, O'Reilly D (2018). Variation of caregiver health and mortality risks by age: A census-based record linkage study. Am. J. Epidemiol..

[32] Lindt N, van Berkel J, Mulder BC (2020). Determinants of overburdening among informal carers: A systematic review. BMC Geriatr..

[33] Haugland BSM, Hysing M, Sivertsen B (2022). Study progress, recreational activities, and loneliness in young adult carers: A national student survey. BMC Psychol..

[34] Wepf H, Joseph S, Leu A (2021). Benefit finding moderates the relationship between young carer experiences and mental well-being. Psychol. Health.

[35] Rose HD, Cohen K (2010). The experiences of young carers: A meta-synthesis of qualitative findings. J. Youth Stud..

[36] Moore T, McArthur M, Morrow R (2009). Attendance, achievement and participation: Young carers’ experiences of school in Australia. Aust. J. Educ..

[37] Gaffney H, Ttofi MM, Farrington DP (2019). Evaluating the effectiveness of school-bullying prevention programs: An updated meta-analytical review. Aggress. Violent Behav..

[38] Fraguas D (2021). Assessment of school anti-bullying interventions: A meta-analysis of randomized clinical trials. JAMA Pediatrics.

[39] Australian Bureau of Statistics. *Disability, Ageing and Carers, Australia: Summary of Findings.*, <https://www.abs.gov.au/statistics/health/disability/disability-ageing-and-carers-australia-summary-findings/latest-release> (2018).

[40] Hunt, G., Levine, C. & Naiditch, L. *Young Caregivers in the U.S.: Findings from a National Survey*, <http://www.caregiving.org/data/youngcaregivers.pdf> (2005).

