# Supplementary material for: The mental health impact of school bullying among young carers in Australia: a causal mediation analysis
Source: Sci Rep. 2023 Oct 5;13:16788. doi: 10.1038/s41598-023-43464-5 (PMC10555989; doi:10.1038/s41598-023-43464-5)
Supplement: Supplementary file 1 — Supplementary Information. [file 41598_2023_43464_MOESM1_ESM.pdf]

The mental health impact of school bullying among young carers in Australia – a causal mediation analysis

Ludmila Fleitas Alfonzo, Ankur Singh, George Disney, Tania King

Supplementary files

Table of Contents

Supplementary Table 1. Bullying victimisation assessment in LSAC wave 7 (child aged 16/17 years)................2

Supplementary Figure 1. Directed acyclic graph...2

Supplementary table 2. Distribution of missing data by exposure status (n=3375)...3

Supplementary table 3. Distribution of parental employment and household income in LSAC waves 5 and 6.....4

Supplementary Table 4. Estimates of natural indirect effects and natural direct effects of the association between informal care and mental health, mediated by bullying victimisation in the imputed sample (n=3,375) 4

Supplementary Table 1. Bullying victimisation assessment in LSAC wave 7 (child aged 16/17 years)

**Item**	**Response options**
Someone hit or kicked me on purpose	Yes/No
Someone grabbed or shoved me on purpose	Yes/No
Someone threatened to hurt me	Yes/No
Someone said mean things to me or called me names	Yes/No
Someone tried to keep others from being my friend	Yes/No
Someone did not let me join in what they were doing	Yes/No
Someone spread rumours about me behind my back	Yes/No
Someone deliberately tried to hurt me by not talking to me	Yes/No
Someone deliberately excluded me from an activity, event or group	Yes/No

Supplementary Figure 1. Directed acyclic graph.

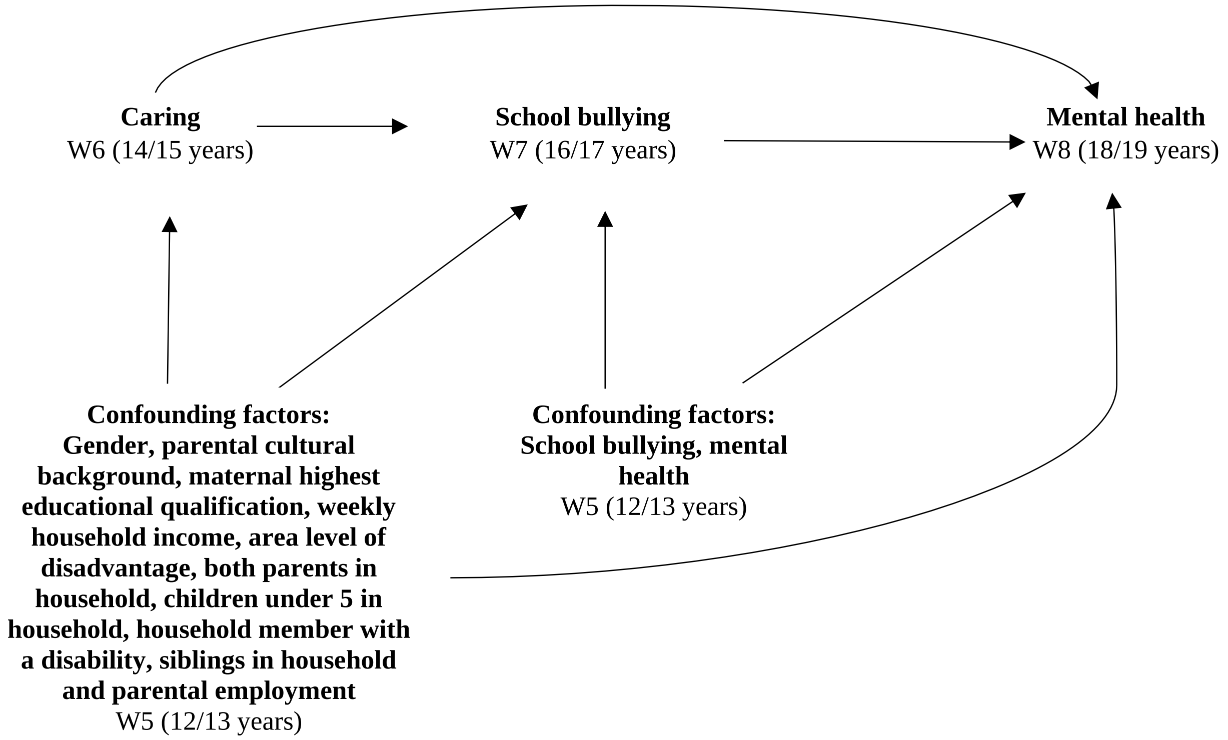

Note: Directed acyclic graph (DAG) for the association between informal caring and mental health mediated through school bullying.

Supplementary table 2. Distribution of missing data by exposure status (n=3375)

	**Non-cares**	**Young carers**		**All**
		**Light**	**Moderate/heavy**	
**Outcome**	n (%)	n (%)	n (%)	n (%)
Kessler 10 scores (mean (SD))	512 (26.3)	278 (27.2)	125 (31.0)	915 (27.1)
**Mediator**				
School bullying (Past month)	278 (14.3)	147 (14.4)	66 (16.4)	491 (14.6)
**Covariates**				
Gender	0	0	0	0
Parental cultural background	106 (5.44)	51 (4.99)	33 (8.19)	68 (2.14)
Maternal highest educational qualification	35 (1.80)	16 (1.56)	11 (2.73)	62 (1.84)
Weekly household income	153 (7.85)	93 (9.09)	39 (9.68)	285 (8.44)
Area level of disadvantage (SEIFA)	36 (1.85)	16 (1.56)	12 (2.98)	64 (1.90)
Both parents in household	36 (1.85)	16 (1.56)	12 (2.98)	64 (1.90)
Children under 5 in household	36 (1.85)	16 (1.56)	12 (2.98)	64 (1.90)
Household member with a disability	36 (1.85)	16 (1.56)	12 (2.98)	64 (1.90)
Siblings in household (mean (SD))	36 (1.85)	16 (1.56)	12 (2.98)	64 (1.90)
Parental employment	297 (15.2)	204 (19.9)	76 (18.9)	577 (17.1)
Prior school bullying	91 (4.67)	40 (3.91)	23 (5.71)	154 (4.56)
Strength and difficulties score (SDQ)	70 (3.59)	28 (2.74)	16 (3.97)	114 (3.38)

Supplementary table 3. Distribution of parental employment and household income in LSAC waves 5 and 6.

Parental employment	Wave 5	Wave 6
	n (%)	*n (%)*
**Both employed**	2193 (78.4)	2203 (79.4)
**One employed**	919 (27.8)	945 (28.1)
**Both unemployed**	193 (5.84)	210 (6.25)
**Weekly household income (quintiles)**		
**1 (lowest)**	583 (18.9)	622 (20.2)
**2**	610 (19.7)	617 (20.1)
**3**	615 (19.9)	597 (19.4)
**4**	631 (20.4)	616 (20.0)
**5 (highest)**	651 (21.1)	623 (20.3)

Supplementary Table 4. Estimates of natural indirect effects and natural direct effects of the association between informal care and mental health, mediated by bullying victimisation in the imputed sample (n=3,375)

	Total effect	Natural direct effect	Natural indirect effect[a]	Proportion mediated
**Light carers**	*ß (95%CI)*	*ß (95%CI)*	*ß (95%CI)*	*Proportion (95%CI)*
Model 1[b]	0.87 (0.32, 1.40)	0.74 (0.17, 1.21)	0.13 (-0.01, 0.22)	0.15 (-0.01, 0.39)
Model 2[c]	0.60 (0.07, 1.05)	0.51 (-0.05, 0.96)	0.10 (-0.01, 0.20)	0.17 (-0.02, 1.65)
Model 3[d]	0.60 (0.07, 1.05)	0.49 (-0.05, 0.96)	0.11 (0.00, 0.21)	0.18 (0.00, 1.71)
**Moderate-to-heavy carers**				
Model 1[b]	1.61 (0.78, 2.47)	1.39 (0.61, 2.21)	0.22 (0.05, 0.45)	0.13 (0.03, 0.31)
Model 2[c]	1.33 (0.68, 2.11)	1.18 (0.50, 1.96)	0.16 (0.02, 0.38)	0.12 (0.02, 0.32)
Model 3[d]	1.34 (0.68, 2.11)	1.12 (0.46, 1.89)	0.22 (0.06, 0.43)	0.17 (0.03, 0.40)

Notes:

[a]Effect mediated through school bullying

[b]Adjusted for gender, parental cultural background, maternal highest educational qualification, weekly household income, area level of disadvantage, both parents in household, children under 5 in household, household member with a disability, siblings in household and parental employment.

[c]Adjusted for variables in model 1 and mental health at 12/13 years (SDQ scores)

[d]Adjusted for variable in model 2 and school bullying at 12/13 years
